# Supplementary material for: Integrative Transcriptomic Analysis Reveals Distinctive Molecular Traits and Novel Subtypes of Collecting Duct Carcinoma
Source: Cancers (Basel). 2021 Jun 10;13(12):2903. doi: 10.3390/cancers13122903 (PMC8230422; doi:10.3390/cancers13122903)
Supplement: Supplementary file 1 [file cancers-13-02903-s001.zip › cancers-1201903-proof-Supplementary Figures.pdf]

cancers MDPI

Supplementary Material: Integrative Transcriptomic Analysis Reveals Distinctive Molecular Traits and Novel Subtypes of Collecting Duct Carcinoma

Chiara Gargiuli, Pierangela Sepe, Anna Tessari, Tyler Sheetz, Maurizio Colecchia, Filippo Guglielmo Maria de Braud, Giuseppe Procopio, Marialuisa Sensi, Elena Verzoni and Matteo Dugo

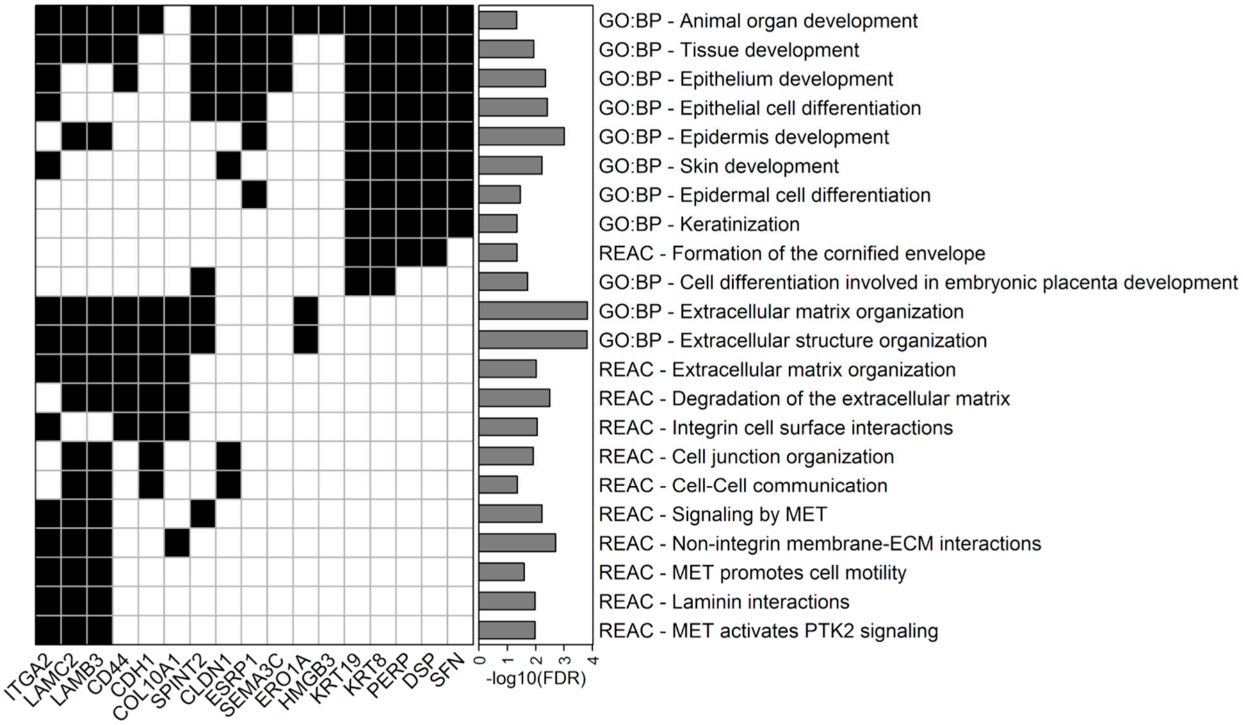

Figure S1. Functional analysis of the CDC-specific gene signature. The heatmap shows overlap between genes of the CDC-specific signature and pathways from different sources that are significantly (adjusted p-value < 0.05) over-represented in the signature.

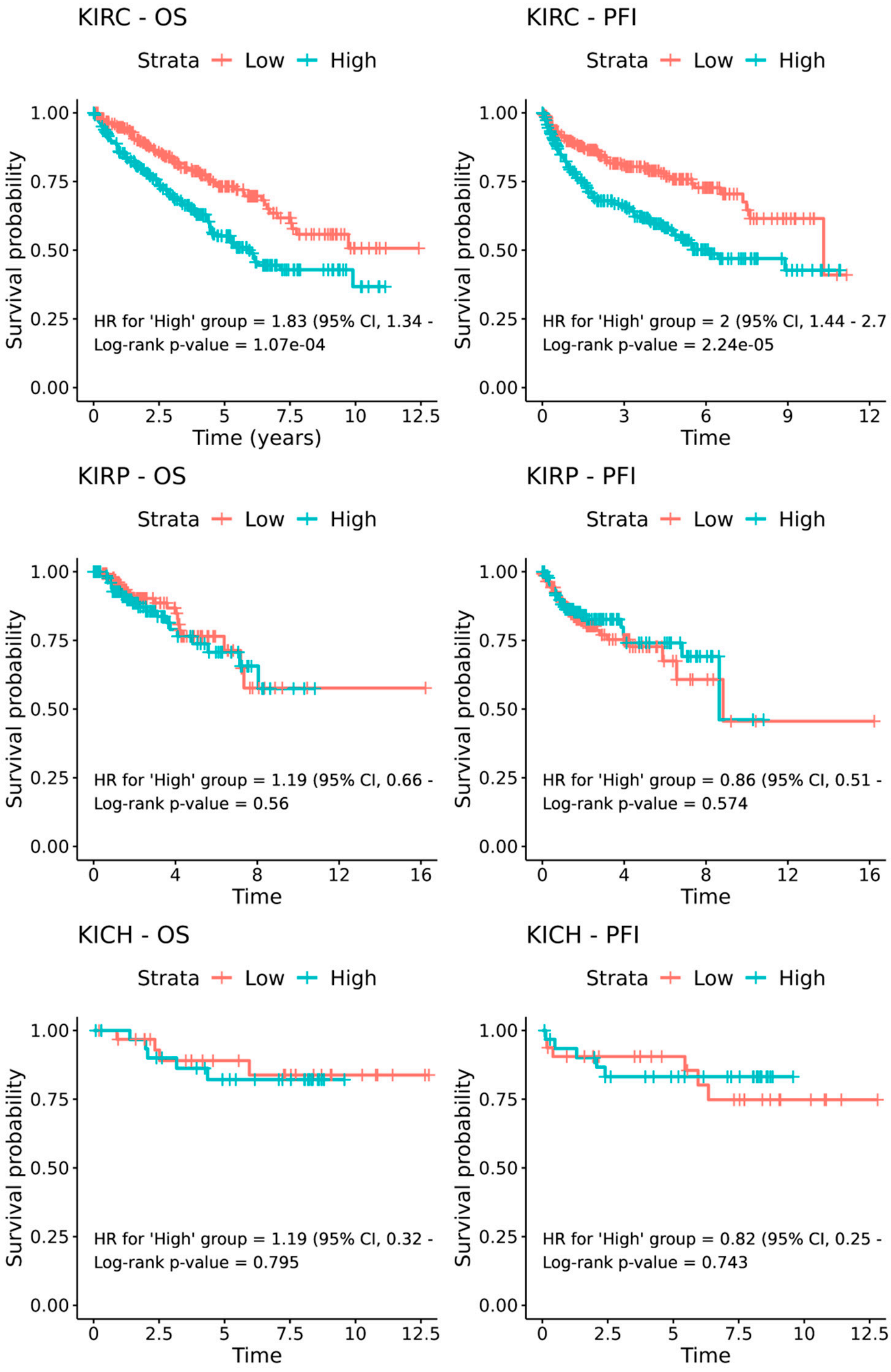

Figure S2. Kaplan-Meier curves for overall survival (OS) and progression-free interval (PFI) of clear cell (TCGA-KIRC), papillary (TCGA-KIRP) and chromophobe (TCGA-KICH) carcinoma patients of TCGA dataset. Patients of each set were stratified in high and low CDC-specific signature expression according to the median score of the signature calculated by singscore.

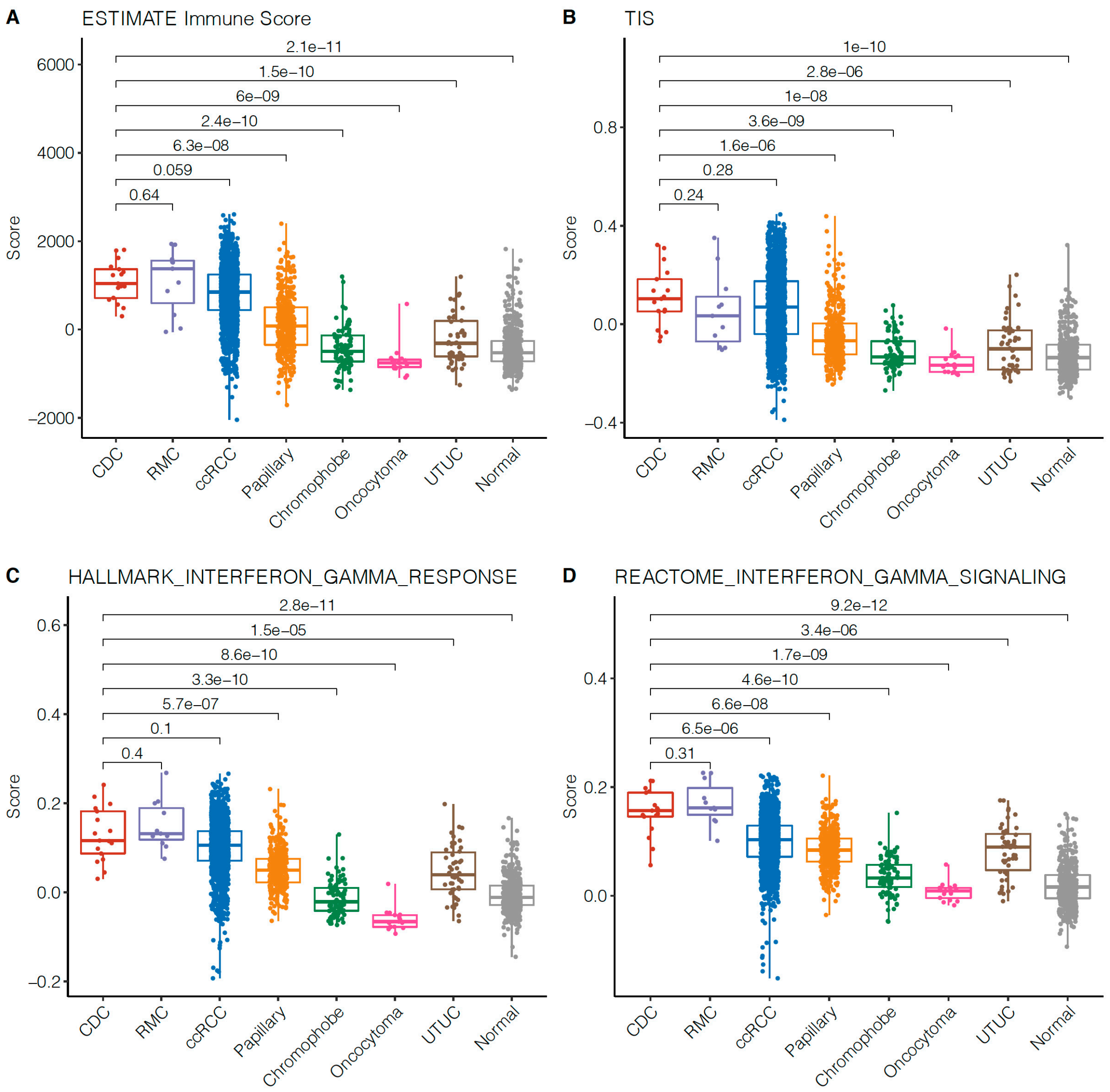

Figure S3. Inference of immune infiltration in kidney cancer histologies. (**A**) Overall immune infiltration score defined by the ESTIMATE algorithm. (**B–D**) Single-sample scores of TIS and interferon-γ related gene sets. *p*-values by Wilcoxon rank-sum test.

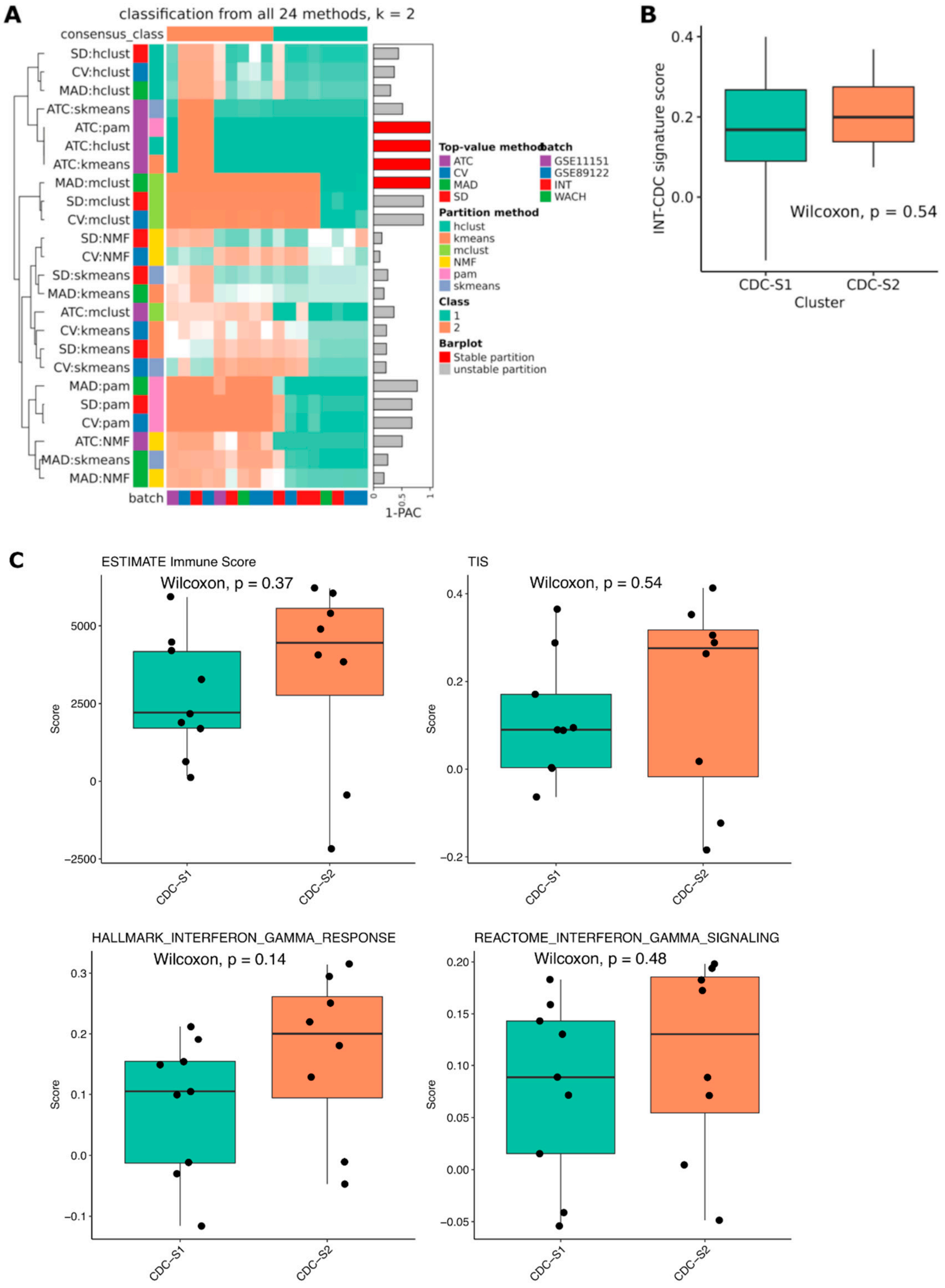

Figure S4. Clustering of CDC tumors. (**A**) Heatmap of the COLA partitions obtained with the 24 combinations of gene variability measures and clustering methods. The bar at the top shows the consensus classification. SD: standard deviation; MAD: median absolute deviation; CV: coefficient of variation; ATC: ability to correlate; NMF: non-negative matrix factorization; pam: partition around medoids. (**B**) Boxplot of the single sample scores of the INT-CDC signature in the two CDC subtypes. (**C**) Boxplots showing the single sample scores of ESTIMATE immune infiltration, TIS, and interferon-γ related gene sets in the two CDC subtypes. *p*-values by Wilcoxon rank-sum test.
